# Molecular prognostic factors for liver transplantation of unresectable metastatic colorectal cancer

**DOI:** 10.1093/bjs/znaf072

**Published:** 2025-04-16

**Authors:** Seyed H Moosavi, Kushtrim Kryeziu, Ina A Eilertsen, Luís Nunes, Merete Hektoen, Barbara Niederdorfer, Henrik M Reims, Trygve Syversveen, Harald Grut, Svein Dueland, Pål-Dag Line, Ragnhild A Lothe, Anita Sveen

**Affiliations:** Department of Molecular Oncology, Institute for Cancer Research, Oslo University Hospital, Oslo, Norway; Department of Molecular Oncology, Institute for Cancer Research, Oslo University Hospital, Oslo, Norway; Department of Molecular Oncology, Institute for Cancer Research, Oslo University Hospital, Oslo, Norway; Department of Molecular Oncology, Institute for Cancer Research, Oslo University Hospital, Oslo, Norway; Department of Molecular Oncology, Institute for Cancer Research, Oslo University Hospital, Oslo, Norway; Department of Molecular Oncology, Institute for Cancer Research, Oslo University Hospital, Oslo, Norway; Department of Pathology, Oslo University Hospital, Oslo, Norway; Department of Radiology and Nuclear Medicine, Oslo University Hospital, Oslo, Norway; Department of Radiology, Vestre Viken Hospital Trust, Drammen, Norway; Transplant Oncology Research Group, Department of Transplantation Medicine, Oslo University Hospital, Oslo, Norway; Transplant Oncology Research Group, Department of Transplantation Medicine, Oslo University Hospital, Oslo, Norway; Institute of Clinical Medicine, Faculty of Medicine, University of Oslo, Oslo, Norway; Department of Molecular Oncology, Institute for Cancer Research, Oslo University Hospital, Oslo, Norway; Institute of Clinical Medicine, Faculty of Medicine, University of Oslo, Oslo, Norway; Department of Molecular Oncology, Institute for Cancer Research, Oslo University Hospital, Oslo, Norway; Institute of Clinical Medicine, Faculty of Medicine, University of Oslo, Oslo, Norway

*To the Editor,*

Liver transplantation (LT) offers a potential for long-term survival in highly selected patients with unresectable liver-confined metastases of colorectal cancer (CRC)^1^. Patient selection criteria for LT are extensive and based on clinicopathological and radiological prognostic factors^2^. However, frequent recurrences after LT and scarcity of donor organs highlight the need for further optimization^3^. There is also a concern that high survival rates after LT can be attributed to indolent disease and selection of patients who might also have done well with conventional treatments^4^. This study addresses these concerns from a molecular biology perspective. Mutations in 20 CRC-relevant genes (*Table S1*) and transcriptomic profiles of liver metastases from 34 patients included in prospective trials of LT for unresectable metastatic CRC at Oslo University Hospital were analysed for prognostic potential and compared with those of resected liver metastases from 98 patients (*Table S2*, *supplementary methods*, *supplementary appendixes*, and *Figs S1–S4*).

The distribution of somatic mutations in the LT cohort (*Fig. 1a* and *Table S3*) was similar to that in the resection cohort (*P* ≥ 0.273; *Fig. S4*) and in a previously published series of unresected liver metastases (73 patients; *P* > 0.176, Fisher’s exact test; *Table S4*). None of the most frequently mutated genes (*APC*, *TP53*, *KRAS*/*NRAS* (*RAS*), or *SMAD4*) was associated with baseline clinicopathological characteristics (*Table S5*) or patient survival in the LT cohort after multiple testing correction (*Table S6*). Co-mutations of *RAS* and *TP53* have prognostic relevance for resectable liver metastases^5^ and were found in 10 patients (29%) in the LT cohort and in 25 patients (26%) in the resection cohort. *RAS*/*TP53* co-mutations were associated with a shorter median overall survival after LT (21 *versus* 60 months; *Fig. 1b* and *Table S6*). The prognostic effect did not appear to be attributed to either *RAS* or *TP53* alone (*Fig. S5*), was stronger in the LT cohort than in the resection cohort (HR 4.0 (95% c.i. 1.7 to 9.3) and 1.7 (95% c.i. 1.0 to 3.0) respectively), and was stronger than or similarly strong to clinicopathological factors previously shown to be prognostic in the LT cohort (*Table S6*, *supplementary results*)^1^. The co-mutations were more frequent in patients with high-risk clinicopathological features, but significant overlap was found only with right-sided primary tumour location (before multiple testing correction; *Table S5*). Furthermore, the co-mutations retained prognostic value in bivariable analyses with each clinicopathological factor (*Table S7*) and the prognostic effect was found mainly among patients in the low-risk clinicopathological subgroups (*Fig. S6*). These data suggest that LT should be carefully considered in patients with *RAS*/*TP53* co-mutations. The main benefit from implementation of this molecular marker would be to exclude from further consideration patients who otherwise have a favourable risk profile and pursue other treatment options to avoid the possible futile use of liver grafts.

**Fig. 1:**
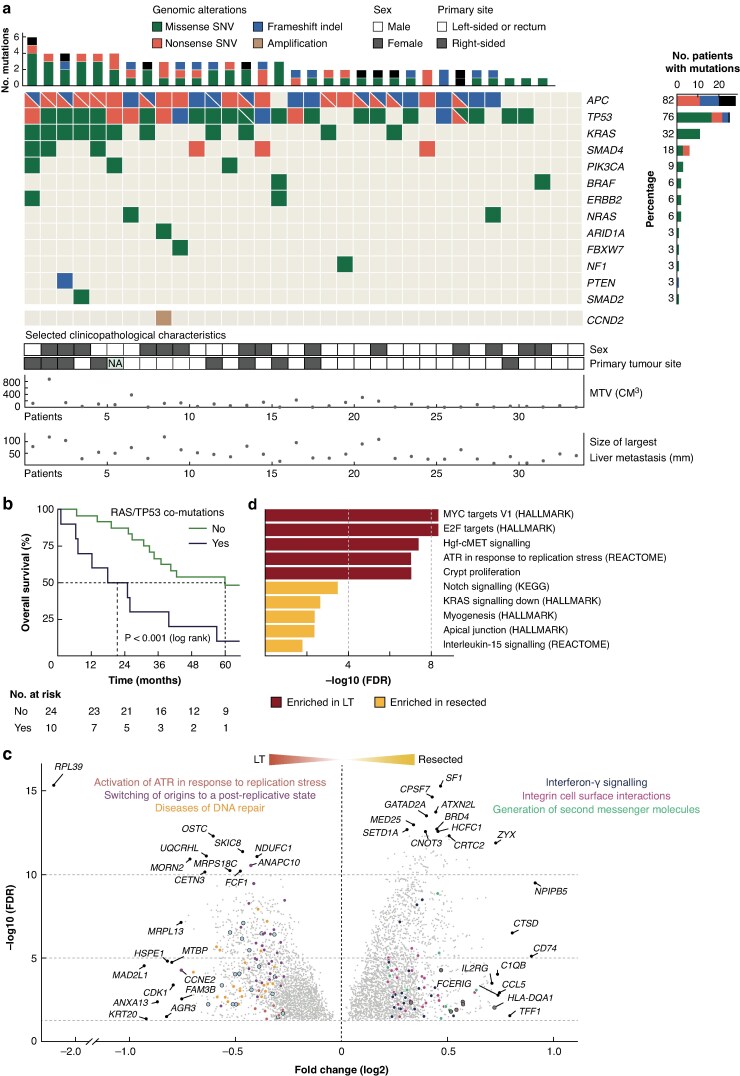
Molecular characteristics of patients treated by LT for metastatic CRC **a** Oncoplot of somatic mutations for each gene in rows (14 genes; ordered according to mutation frequency) and each patient in the LT cohort in columns (34 patients; ordered according to the number of mutated genes). The mutation type is indicated by colour keys. Bar plots summarize the number of mutations per patient (top) and gene (right). Cells split by diagonal lines in the oncoplot and coloured black in the bar plots indicate genes and tumours with two mutations (counted once per tumour). Selected clinicopathological characteristics are indicated in the panels below. **b** Kaplan–Meier plot of overall survival according to *RAS*/*TP53* co-mutations in patients treated by LT (34 patients). **c** Volcano plot of differentially expressed genes between liver metastases in the LT cohort (34 patients) and in the resection cohort (98 patients). The top 20 up-regulated genes in each group are indicated by gene symbols (ranked by log2 fold change and *P* value corrected for the FDR, plotted on log10 scale). The coloured dots indicate genes included in the top three enriched pathways from over-representation analysis of the REACTOME database, as specified (ranked by statistical significance). Black outlines indicate genes included in more than one of the pathways. **d** Bar plot of the five most significantly enriched signatures in the LT cohort (34 patients) and in the resection cohort (98 patients) from gene set enrichment analysis of a custom gene set collection (260 gene sets; *Table S10*). *P* values are FDR-corrected and plotted on log10 scale. SNV, single nucleotide variant; indel, insertion and deletion; MTV, metabolic tumour volume on PET; FDR, false discovery rate; LT, liver transplantation; CRC, colorectal cancer.

Differential gene expression analysis showed subtle differences between the LT cohort and the resection cohort, with low fold changes of all genes (<1 on log2 scale), except *RPL39* (*Fig. 1c* and *Tables S8*, *S9*). However, gene set enrichment analysis indicated a more proliferative phenotype in the LT cohort (*Fig. 1d* and *Table S10*). This was supported by comparison of a transcriptomic proliferative index and was consistent with the more extensive disease burden in the LT cohort (*Table S2*, *supplementary results, and Figs. S7, S8*). This alleviates the concern that the patients selected for LT had indolent and less aggressive disease than the patients selected for liver resection with respect to molecular tumour biology.

This study supports a role for molecular tumour profiling in personalized treatment decisions in transplant oncology of metastatic CRC. A major limitation was the low number of patients. However, small sample size is a challenge in most transplant oncology studies and the prognostic value of *RAS*/*TP53* co-mutations also among patients treated by liver resection^5^ should further motivate new studies in the unique context of LT^3^.

## Supplementary Material

znaf072_Supplementary_Data

## Data Availability

The data sets generated and analysed in the present study are publicly available in repositories or in the *Supplementary material* of the manuscript. Gene expression data for the liver transplantation cohort have been deposited in the Gene Expression Omnibus of the National Center for Biotechnology Information (NCBI) under accession code GSE282186 (https://www.ncbi.nlm.nih.gov/geo/query/acc.cgi?acc=GSE282186). Resected liver metastasis samples have previously been deposited under accession code GSE159216 (https://www.ncbi.nlm.nih.gov/geo/query/acc.cgi?acc=GSE159216). Mutation data are available in *Table S3*.
