# Supplementary material for: Molecular prognostic factors for liver transplantation of unresectable metastatic colorectal cancer
Source: Br J Surg. 2025 Apr 16;112(4):znaf072. doi: 10.1093/bjs/znaf072 (PMC12000646; doi:10.1093/bjs/znaf072)
Supplement: znaf072_Supplementary_Data [file znaf072_supplementary_data.docx]

Molecular prognostic factors for liver transplantation of unresectable metastatic colorectal cancer

Seyed H. Moosavi^1^, Kushtrim Kryeziu^1^, Ina A. Eilertsen^1^, Luís Nunes^1^, Merete Hektoen^1^, Barbara Niederdorfer^1^, Henrik M. Reims^2^, Trygve Syversveen^3^, Harald Grut^4^, Svein Dueland^5^, Pål-Dag Line^5,6^, Ragnhild A. Lothe^1,6^, Anita Sveen^1,6^

**Affiliations:** ^1^Department of Molecular Oncology, Institute for Cancer Research, Oslo University Hospital, Oslo, Norway; ^2^Department of Pathology, Oslo University Hospital, Oslo, Norway; ^3^Department of Radiology and Nuclear Medicine, Oslo University Hospital, Oslo, Norway, ^4^Department of Radiology, Vestre Viken Hospital Trust, Drammen, Norway, ^5^Transplant Oncology Research Group, Department of Transplantation Medicine, Oslo University Hospital, Oslo, Norway; ^6^Institute of Clinical Medicine, Faculty of Medicine, University of Oslo, Oslo, Norway

**Corresponding author**: Anita Sveen, Department of Molecular Oncology, Institute for Cancer Research, Oslo University Hospital, P.O. box 4953 Nydalen, NO-0424 Oslo, Norway; ORCID: 0000-0001-8219-6251

**Supplementary Materials – Index**

[Supplementary Methods 4](#_Toc192259258)

[Patient materials 4](#_Toc192259259)

[Gene expression analyses 5](#_Toc192259260)

[Tumor purity and sample selection 5](#_Toc192259261)

[Computational deconvolution to account for tumor purity 6](#_Toc192259262)

[Gene panel sequencing 7](#_Toc192259263)

[Statistical Analyses 9](#_Toc192259264)

[Supplementary Results 9](#_Toc192259265)

[Prognostic value of *RAS*/*TP53* co-mutations not confounded by *BRAF*^V600E^ 9](#_Toc192259266)

[Molecular similarity of *RAS*/*TP53* co-mutated tumors and tumors with high MTV 9](#_Toc192259267)

[Proliferative phenotype not attributed to younger patient age 9](#_Toc192259268)

[Supplementary Appendixes 11](#_Toc192259269)

[Supplementary Figures and Tables 12](#_Toc192259270)

[Figure S1. Transcriptomic comparison of sample types. 12](#_Toc192259271)

[Figure S2. Hepatocyte expression scores according to sample type. 13](#_Toc192259272)

[Figure S3. Evaluation of computational deconvolution approach. 14](#_Toc192259273)

[Figure S4. Mutation distribution in patients treated by LT and liver resection. 15](#_Toc192259274)

[Figure S5. Overall survival according to RAS and TP53 mutation status. 17](#_Toc192259275)

[Figure S6. Overall survival of patients treated by liver transplantation (n = 34) according to RAS/TP53 co-mutations and stratified by clinicopathological variables. 19](#_Toc192259276)

[Figure S7. Transcriptomic proliferative index of the LT (n = 34) and resection cohorts (n = 98). 20](#_Toc192259277)

[Figure S8. Gene set enrichment analysis of the LT cohort (n = 34). 21](#_Toc192259278)

[Table S1. Overview of the custom gene panel for DNA sequencing 22](#_Toc192259279)

[Table S2. Baseline characteristics of the patient cohorts 23](#_Toc192259280)

[Table S3. Detected mutations in the LT and resection cohorts 25](#_Toc192259281)

[Table S4. Comparison of mutation frequencies between the external dataset of biopsies from unresected liver metastases (MSK; n = 73)^27^ and LT cohort (n = 34) 26](#_Toc192259282)

[Table S5. Selected clinicopathological variables according to mutation status of the most frequently mutated genes among patients in the LT cohort (n = 34) 27](#_Toc192259283)

[Table S6. Univariable Cox proportional hazards analyses of OS among patients in the LT cohort (n = 34) 28](#_Toc192259284)

[Table S7. Bivariable Cox proportional hazards analyses for *RAS*/*TP53* co-mutations and selected variables among patients in the LT cohort (n = 34) 29](#_Toc192259285)

[Table S8. Differentially expressed genes (FDR-corrected P-value < 0.05) between patients in the resection (n = 98) versus LT cohort (n = 34) 30](#_Toc192259286)

[Table S9. Top 10 over-represented pathways in the Reactome database (ranked by statistical significance) for differentially expressed genes between the resection (n = 98) and LT cohorts (n = 34)^a^ 31](#_Toc192259287)

[Table S10. Gene set enrichment analyses (GSEA) of sample groups 32](#_Toc192259288)

[References 33](#_Toc192259289)

# Supplementary Methods

## Patient materials

Patients were included from prospective nonrandomized controlled trials of liver transplantation (LT) for unresectable metastatic colorectal cancer (CRC) confined to the liver at Oslo University Hospital, Norway between November 2006 and November 2020. Fresh-frozen tumor tissue and adjacent non-malignant samples from liver explants were available from 48 patients in the SECA-I/II and RAPID studies (Table S2). The patient inclusion and exclusion criteria, follow-up and clinicopathological prognostic factors for long-term survival have previously been reported^1,2^. Patients received chemotherapy while on the organ transplant wait list, and all sampled tumors were exposed to chemotherapy. No patients received adjuvant chemotherapy after LT, and recurrences were treated according to standard protocols as previously described^3^. Immunosuppressive treatment was given according to study protocol^4^.

Patients in the resection cohort were included from an ongoing observational study of liver resection for metastatic CRC according to standard protocols at Oslo University Hospital^5^. The study involves prospective biobanking of fresh-frozen samples of all metastatic lesions larger than 5 mm and adjacent non-malignant tissue in the resected specimens^6^. Patients in the current study (n = 99) were included between October 2013 and March 2018, and were selected to match specific features of the LT cohort, including exposure to neoadjuvant chemotherapy and liver-only metastases (no apparent extra-hepatic disease). Patients in the resection cohort had a lower number of liver lesions and were older than patients in the LT cohort, while there was no difference in the distribution of other clinicopathological characteristics (Table S2). Selected analyses were repeated with a subset of patients in the resection cohort (n = 40) chosen to match the mean and median age of the LT cohort. A single liver metastasis sample from each patient in the resection cohort and non-malignant liver tissue from 19 of the patients were included for analyses.

RNA and DNA were extracted using the Allprep DNA/RNA/miRNA Universal kit according to the manufacturer’s instructions (Qiagen GmBH, Hilden, Germany).

The study was conducted in accordance with the Declaration of Helsinki and all patients provided written informed consent. The study has been approved by the Norwegian Data Protection Authority and Regional Committee for Medical and Health Research Ethics, South-Eastern Norway (REC numbers 1.2005.1629, 2010/1805, 2017/780, S-05409, 2012/1392, 2013/580, 2016/1507).

## Gene expression analyses

All samples were analyzed for gene expression on the Human Transcriptome Array 2.0 using 100 ng of total RNA as input and following the manufacturer’s protocol (Thermo Fisher Scientific, Waltham, MA, USA). The data for resected liver metastases have previously been published^7,8^. Raw intensity CEL data files were preprocessed according to the robust multi-array average method without normalization, and with a custom chip definition file from Brainarray^9^ (hta20_Hs_ENTREZG version 25.0) using the justRMA function in the R package affy (version 1.80.0)^10^. Entrez IDs were mapped to HUGO Gene Nomenclature Committee symbols using the org.Hs.eg.db package (version 3.18) from Bioconductor. Gene annotations were retrieved using the R package biomaRt (version 2.60.1)^11^, and only protein-coding genes were included for analyses (n = 18 765). Quantile normalization was applied to the filtered expression dataset using the normalizeBetweenArrays function in the R package limma (version 3.58.1)^12^.

Differential gene expression analysis was performed by empirical Bayes estimation and correction for the false discovery rate (FDR) according to the Benjamini-Hochberg method using the R package limma. Over-representation analysis of differentially expressed genes among Reactome^13^ gene sets and FDR correction was performed with the enrichPathway function in the R package ReactomePA (version 1.48.0; the minimum and maximum gene set size was set to 30 and 100 genes, respectively). Gene set enrichment analysis of a custom gene set collection (n = 260; list of gene sets in Table S10) between sample groups was conducted using the camera function in the limma package with FDR correction. Single-sample enrichment scores were estimated with gene set variation analysis using the gsva function in the R package GSVA (version 1.50.0)^14^. The proliferative index was calculated as the median expression value of genes specified in the R package ProliferativeIndex (version 1.0.1)^15^.

Dimensionality reduction by principal component analysis (PCA) was performed with the prcomp function in the R package stats, and by uniform manifold approximation and projection (UMAP) using the umap function in the R package uwot (version 0.1.16.9, https://github.com/jlmelville/uwot) on the genes (n = 2 000) with largest standard deviation of expression among samples. Annotations of oncogenes and tumor suppressor genes were retrieved from the OncoKB ([https://www.oncokb.org](https://www.oncokb.org/)) and TSGene [([https://bioinfo.uth.edu/TSGene](https://bioinfo.uth.edu/TSGene/)/](https://www.oncokb.org/)) databases (accessed September 9, 2024).

## Tumor purity and sample selection

Tumor purity of the liver metastasis samples was evaluated by comparison of gene expression data from different tissue types, all analyzed on the Human Transcriptome Array 2.0. This included in-house data from colorectal liver metastases (n = 48 and 99 in the LT and resection cohorts, respectively), adjacent non-malignant liver tissue from the resection cohort (n = 19), and primary colorectal tumors (n = 128 stage III-IV cancers; previously published and available from NCBI’s Gene Expression Omnibus [GEO] with accession numbers GSE79959, GSE96528, GSE139170, and GSE178120)^8,16^, as well as a publicly available dataset of adjacent non-malignant liver tissue samples from patients with hepatocellular carcinoma or cholangiocarcinoma (n = 151; downloaded from the GEO with accession number GSE76297)^17^.

All samples were preprocessed and normalized as one dataset, and sample type comparisons were performed by UMAP and PCA. UMAP analysis showed separation of samples according to the tissue of origin along the first dimension (Figure S1). The primary tumors and non-malignant liver tissue samples were separated farthest apart, while the liver metastases from the LT and resection cohorts showed considerable overlap with each other and to some extent with the primary tumors. However, a subset of the metastases (n = 15) clustered with the non-malignant liver samples, indicating low tumor purity. Single-sample enrichment scores (GSVA scores) for a signature of hepatocyte marker genes retrieved from the Human Protein Atlas^18^ (version 23.0; Table 1 in <https://www.proteinatlas.org/humanproteome/tissue/liver> [detected in a single tissue AND enriched in the liver], accessed September 2023; n = 29 of the 30 genes were annotated with HUGO symbols and analyzed) confirmed high hepatocyte scores among the liver metastases that clustered with non-malignant samples (Figure S2). Cryosections of 13 of the 15 samples were stained with hematoxylin and eosin and evaluated by a pathologist. This confirmed no/low tumor cell content and/or necrotic tissue, and all 15 samples were excluded from downstream analyses, including 14 samples in the LT cohort and 1 sample in the resection cohort. There were no significant differences of baseline clinicopathological characteristics between the included and excluded patients in the LT cohort, except a higher liver metabolic tumor volume (MTV) on fluorodeoxyglucose positron emission tomography among included patients (Table S2).

## Computational deconvolution to account for tumor purity

The hepatocyte expression scores varied substantially among the included liver metastasis samples (n = 34 and 98 in the LT and resection cohorts, respectively), indicating variation in sample tumor purity and infiltration with cellular constituents of the liver microenvironment (Figure S2). Consistently, PCA showed a strong positive correlation between PC1 values and the hepatocyte score in both liver metastasis cohorts, but not among primary tumors (low hepatocyte scores) or non-malignant liver samples (high hepatocyte scores; Figure S2A). The hepatocyte score was significantly higher in the LT cohort relative to the resection cohort (Figure S2B), indicating a need to adjust the gene expression dataset prior to comparisons between the cohorts. For this, we adopted a previously used approach for computational deconvolution^8^. In short, the hepatocyte marker genes (n = 29) were used as predictor variables in a linear regression model of the combined liver metastasis dataset (n = 132). This regression model quantified the liver-specific background expression in the samples by summing and normalizing the expression of the hepatocyte markers, followed by a linear regression correction. The residuals from this model represented transformed gene expression data with less influence from the liver-specific background. The R code for this model is included in the Supplementary Appendixes.

To evaluate the approach, the 20 genes with the strongest contribution to the variance explained by PC1 in the liver metastasis dataset before and after adjustment were identified using the facto_summarize function in the R package factoextra (version 1.0.7; Figure S3A). Prior to the adjustment, genes contributing to the variation captured by PC1 had high tissue specificity for the liver, and represented genes involved in the metabolic activity of hepatocytes. After the adjustment, there was a transition towards genes with intestinal epithelial cell functions (according to annotations in the Human Protein Atlas), indicating specificity for colorectal tissue. This indicated that the transformed gene expression dataset was more appropriate for comparisons of CRC-related features between liver metastases in the LT and resection cohorts. Furthermore, the two cohorts had overlapping sample distributions in PCA after adjustment (Figure S3B). All subsequent analyses were performed on the transformed gene expression dataset.

## Gene panel sequencing

Paired tumor and non-malignant tissue samples in the LT cohort (n = 34) and tumor samples in the resection cohort (n = 98) were sequenced for a custom panel of 20 genes selected based on mutation frequency and importance in CRC (Table S1). Sequencing libraries were generated from 50 ng genomic DNA using the Twist Bioscience Library Preparation EF Kit and target enrichment workflow with DNA purification beads following the manufacturer’s protocols (Twist Bioscience, San Francisco, CA, USA). Sequencing was performed with paired-end reads (2×73 nucleotides) on the Illumina MiniSeq system using the MiniSeq High Output Kit (150-cycle; Illumina, San Diego, CA, USA) to a median coverage of 526X and 502X for tumor samples in the LT and resection cohorts, respectively, and 613X for matched normal samples in the LT cohort.

Raw sequencing reads were quality controlled with FastQC (version 0.11.8, https://www.bioinformatics.babraham.ac.uk/publications.html) and aligned to the human reference genome hg38 using the Burrows-Wheeler Alignment tool (BWA, version 0.7.17)^19^. Sequence Alignment Map (SAM) files were converted to Binary Alignment Map (BAM) files, sorted and indexed using Picard (version 2.19.0, <http://broadinstitute.github.io/picard/>). Duplicate reads were identified and removed using the MarkDuplicates function from the Picard tools implemented in GATK^20^ (version 4.1.2.0). Base quality recalibration was performed on the aligned reads using the BaseRecalibrator and ApplyBQSR commands in GATK. Variants were called with MuTect2^21^ and annotated with ANNOVAR^22^ (version 2016Feb01). The candidate mutation list was filtered using a total coverage threshold of at least 15X, a minimum of 5X coverage for the mutant allele, and at least 5% of reads supporting the mutation. Mutations in the LT cohort were determined to be somatic using the patient-matched non-malignant sample as reference. Up to 3 mutant alleles were allowed in the normal sample if the mutant allele fraction was less than 1.5%. In the resection cohort, matched normal samples were not sequenced, and putative germline variants were filtered if reported in the 1000 Genomes Project^23^ with a mutant allele fraction above 1% and/or reported in the Single-Nucleotide Polymorphism database (dbSNP)^24^ but not in COSMIC^25^. Furthermore, only mutations marked with “PASS” or “clustered_events” from Mutect2 were kept. For comparison of mutation frequencies between the LT and resection cohorts, candidate mutations in the LT cohort were filtered using the same criteria (not using the matched normal samples). The overall concordance between the paired-sample and tumor-only filtering approach was 0.92 (95% confidence interval 0.85-0.96; Table S3, Figure S4B). Nonsynonymous exonic single nucleotide variants (missense, nonsense, stoploss) and frameshift indels were considered non-silent. Splice site mutations were excluded due to uncertainty caused by the gene panel design.

Amplifications of the 5 genes covered by probes in both exonic and selected intronic regions (*CCND2*, *EGFR*, *ERBB2*, *MDM2*, and *MYC*) were scored in the LT cohort. The total sequencing coverage of each amplicon in each sample was assessed with the function DepthOfCoverage in GATK (version 3.6). To account for variation in coverage across samples, normalization was performed by dividing the total read counts per amplicon by the sum of reads from both the tumor and matched normal sample. Copy number ratios were calculated by dividing the normalized tumor by normalized normal read counts per amplicon. Gene-level copy number aberrations were summarized as the weighted averages of amplicon-level copy number ratios. Copy number aberrations with P < 0.01 (adjusted for false discovery rate [FDR] using Benjamini-Hochberg) were considered significant. Low-level and high-level amplifications were scored in tumor samples with 5 additional copies (equivalent to a copy number ratio > 3.5) and 15 additional copies (copy number ratio > 8.5) of the gene, respectively.

Mutation data from biopsies of liver metastases and corresponding clinical data for 73 patients with metastatic CRC not treated by metastasectomy were accessed from cBioportal^26^ with the R package cbioportalR (version 1.1.0) and from <https://bit.ly/4bT3E6Q>, respectively^27^.

## Statistical Analyses

All statistical analyses and visualizations were performed using R (version 4.4.1). Mutation frequencies and the distribution of clinicopathological characteristics were compared between patient cohorts by the Fisher's exact test, Pearson’s chi-squared test, and Wilcoxon rank-sum test. Multiple testing adjustments were performed by FDR correction according to the Benjamini-Hochberg method. Survival analyses were conducted with overall survival as the endpoint, estimated from the date of LT, and using the Kaplan-Meier method and Cox proportional hazard models as implemented in the survival package (version 3.7). The proportional hazards assumption was tested using the cox.zph function. Survival plots were generated with the package survminer (version 0.4.9). All statistical tests were two-sided and P-values or FDR-corrected P-values < 0.05 were considered significant.

# Supplementary Results

## Prognostic value of *RAS*/*TP53* co-mutations not confounded by *BRAF*^V600E^

Two patients in the LT cohort had *BRAF*^V600E^ mutations (Figure 1A). These patients were included in a study of extended criteria for LT (SECA-II arm D)^28^. The prognostic effect of the *RAS*/*TP53* co-mutations was retained in analysis of patients with wild-type *BRAF* (n = 32; hazard ratio 4.2, 95% confidence interval 1.8-10.0, P = 0.001 by Cox proportional hazards analysis).

## Molecular similarity of *RAS*/*TP53* co-mutated tumors and tumors with high MTV

Gene set enrichment analyses suggested that tumors with *RAS*/*TP53* co-mutations had similar transcriptomic profiles to tumors with a high MTV (above 70 cm^3^). Both subgroups were enriched with signatures of MYC targets, cell-cycle progression, and proliferative characteristics relative to tumors without the co-mutation or a low MTV, respectively (Figure S8, Table S10). However, there was no significant overlap of tumors with the two features (odds ratio 3.7, 95% confidence interval 0.6-28.2, P=0.1, Fisher’s exact test; Table S5).

## Proliferative phenotype not attributed to younger patient age

Patients in the LT cohort were younger than in the resection cohort (P = 0.002, Fisher’s exact test of groups dichotomized at the median age of the LT cohort; Table S2). However, the transcriptomic proliferative index^15^ was not correlated to patient age across the two cohorts (Figure S7; Spearman correlation -0.1, P = 0.1). Furthermore, gene set enrichment analysis between the LT and resection cohorts based on a subset of patients in the resection cohort (n = 40) selected to match the age distribution of the LT cohort showed highly similar results to the overall cohort (Table S10), supporting that the proliferative phenotype of the LT cohort was not attributed to the younger patient age.

# Supplementary Appendixes

**R code for computational deconvolution approach.**

# Function to perform liver-background adjustment of gene expression data based on
# liver markers
liver_adjustment <- function(expression_data, liver_markers) {
 # Center the gene expression data matrix by
 # subtracting the row means
 centered_expression_data <- sweep(expression_data, 1,
 rowMeans(expression_data), FUN = "-")

# Create a binary indicator variable for liver markers: 1 if the gene is a liver
# marker, 0 otherwise
 liver_marker_indicator <- ifelse(rownames(centered_expression_data) %in%
 liver_markers, 1, 0)

# Calculate the predictor for the linear model as the column sums of liver marker
# expressions normalized to the [0, 1]
 predictor <- colSums(centered_expression_data[liver_marker_indicator ==
 1, ])
 normalized_predictor <- (predictor - min(predictor))/(max(predictor) -
 min(predictor))

# Perform a linear regression to remove the liver-specific background signal from the
# centered gene expression data
 fit <- lm(t(centered_expression_data) ~ normalized_predictor)

# Extract the residuals from the linear model fit which represent the liver-adjusted gene expression data
 adjusted_expression_data <- t(fit$residuals)

# Adjust the gene expression data to ensure all values are non-negative by adding the # absolute value of the minimum adjusted expression data
 adjusted_expression_data <- adjusted_expression_data +
 abs(min(adjusted_expression_data))

# Return the adjusted gene expression data
 return(adjusted_expression_data)
}

# Supplementary Figures and Tables


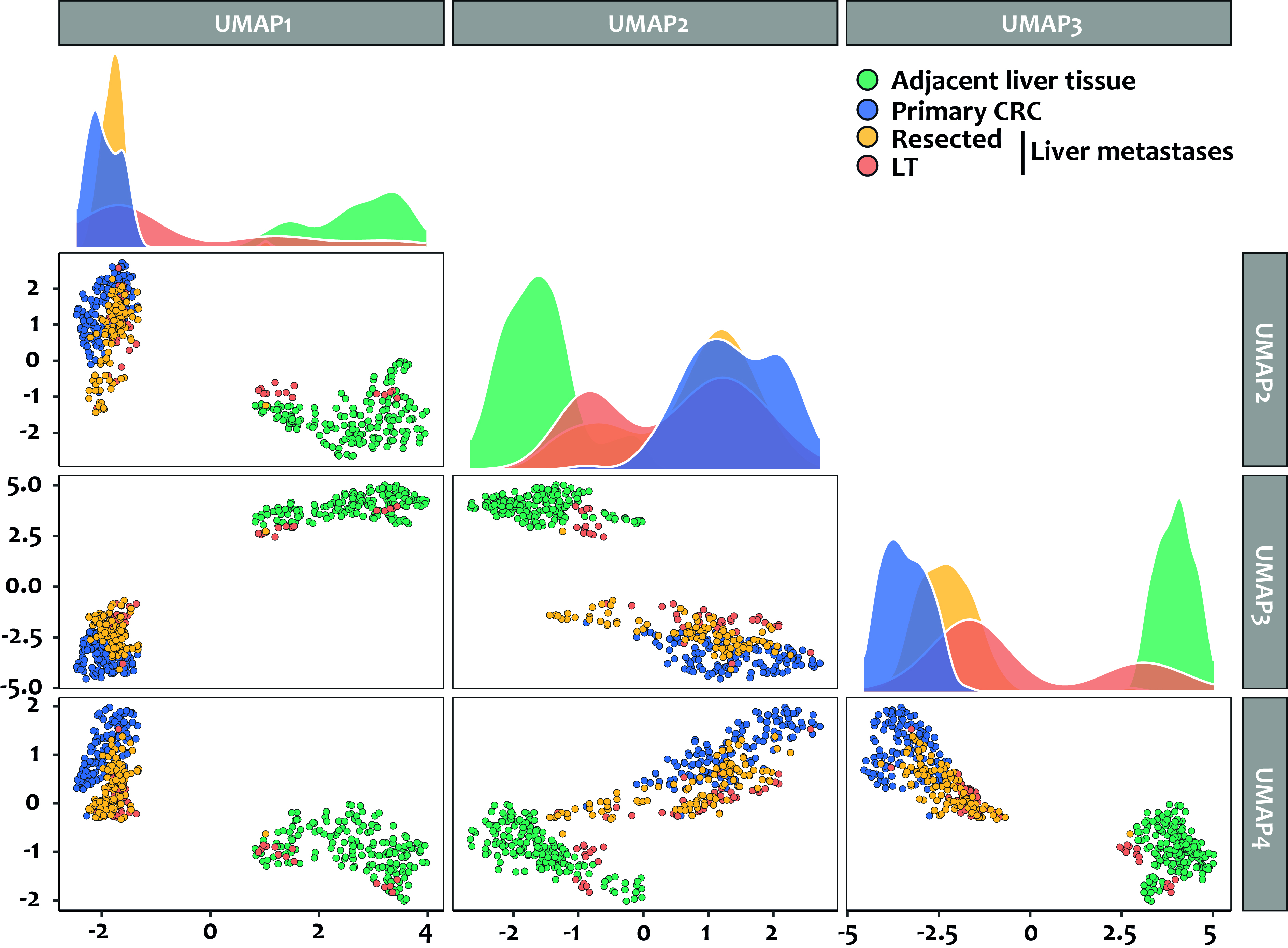


**Figure S1. Transcriptomic comparison of sample types.** Uniform manifold approximation and projection (UMAP) plot of individual samples along the first four dimensions (UMAP1-4) based on genes (n = 2 000) with largest standard deviation of expression across the dataset. Samples are colored according to sample type. Density plots indicate the sample distribution along each of the first three dimensions.


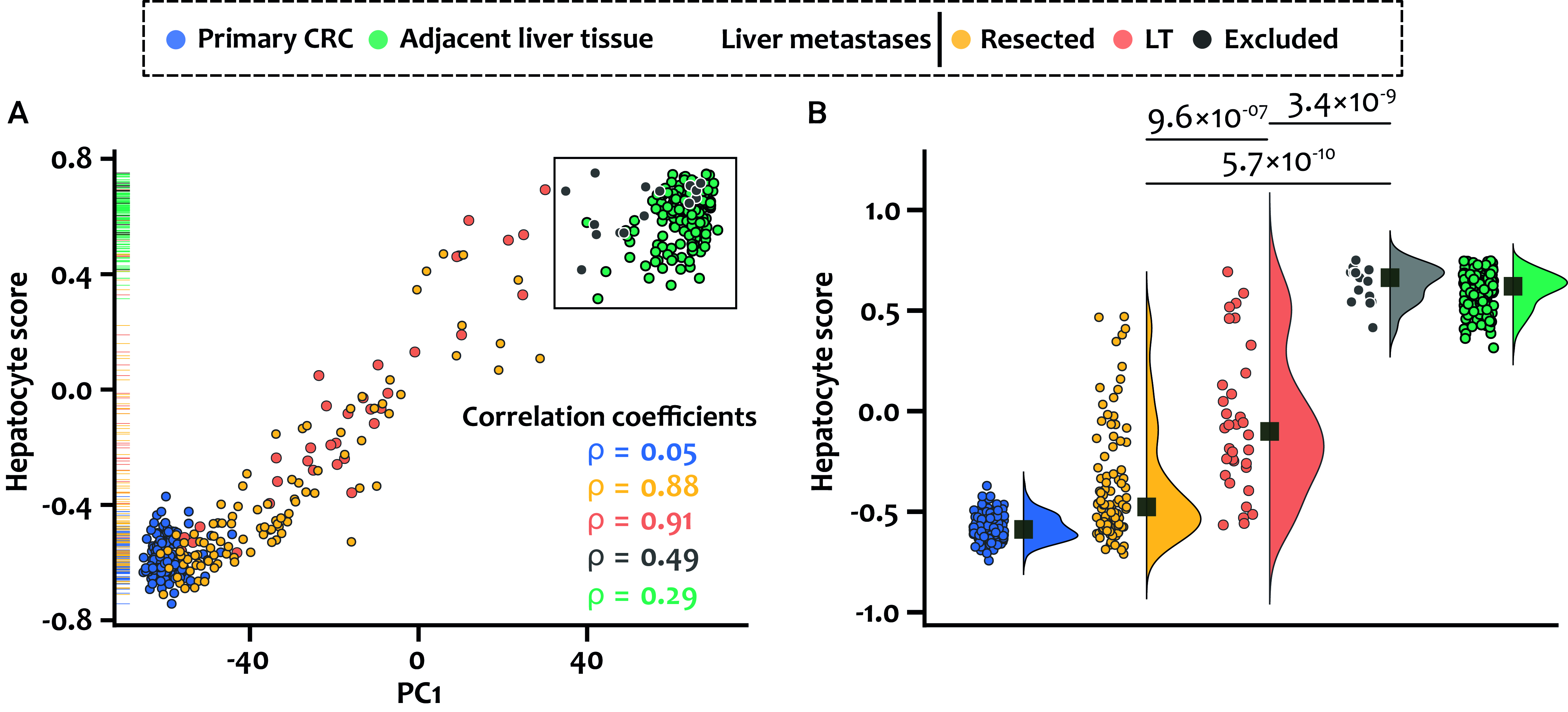


Figure S2. Hepatocyte expression scores according to sample type. A) Comparison of the hepatocyte score from single-sample enrichment analysis of hepatocyte marker genes (n = 29) and the first component (PC1) from PCA of the genes (n = 2 000) with largest standard deviation of expression across all samples. Samples are colored according to sample type, and liver metastases excluded based on low tumor purity are colored dark grey and marked by the black rectangle. Spearman correlation coefficients (ρ) between the hepatocyte scores and PC1 values are given for each sample group. B) Violin plots of the hepatocyte scores according to sample type. P-values are from Wilcoxon rank-sum tests between sample groups as indicated.


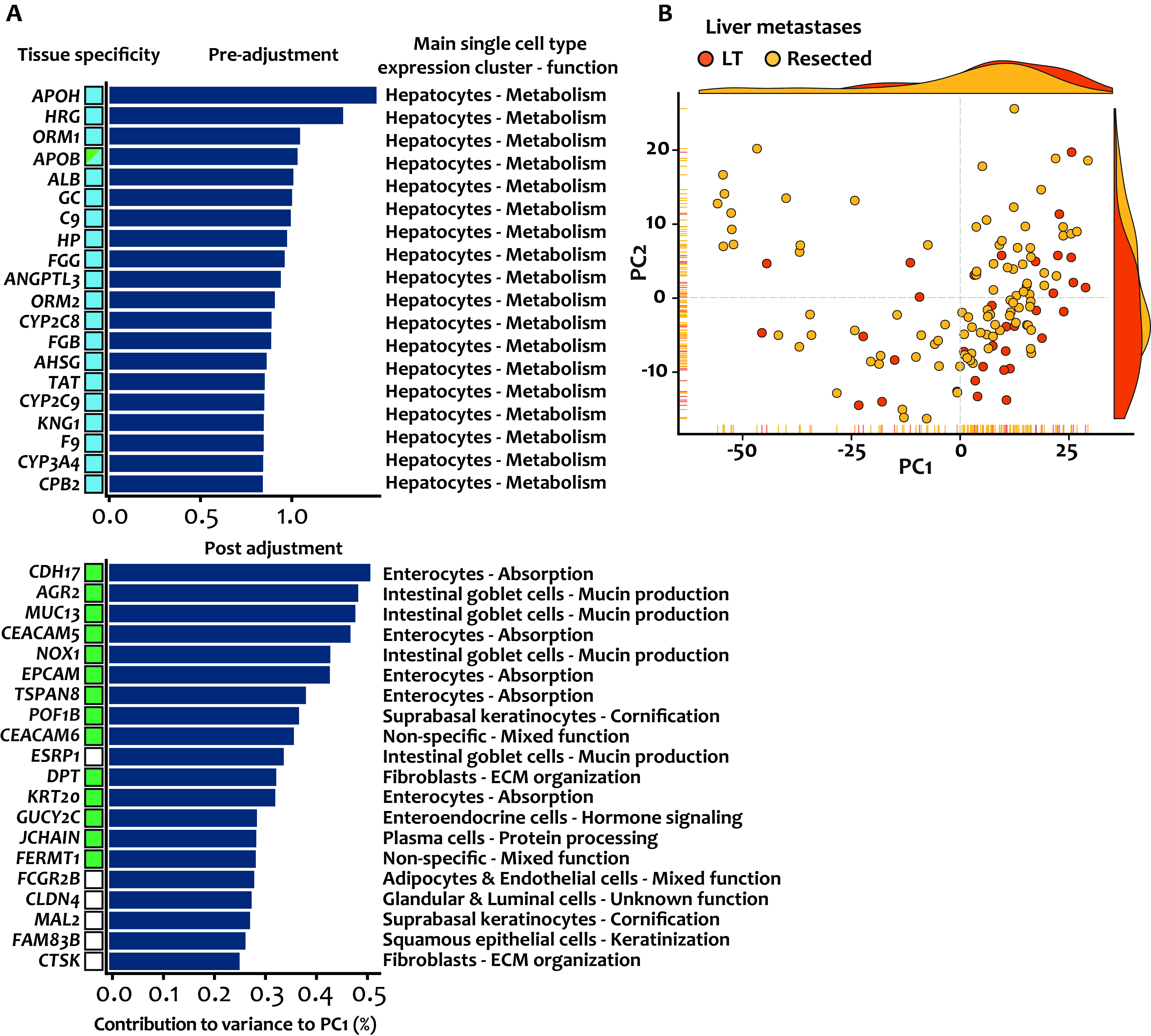


**Figure S3. Evaluation of computational deconvolution approach.** A) Bar graphs of the estimated contribution of each of the top scoring genes (n = 20) to the expression variance captured by the first principal component (PC1) in the combined dataset of liver metastases from the transplantation and resection cohorts (n = 132) before (top) and after (bottom) adjustment for liver background expression. The contribution was calculated as the squared loadings of each gene from PCA normalized by the total variance of PC1. The colored squares indicate tissue-specificity of the genes according to annotations in the Human Protein Atlas (green: intestine; blue: liver; white: other). The main single cell type expression cluster is indicated as denoted according to the “Tissue RNA expression” data in the Human Protein Atlas (for example, www.proteinatlas.org/ENSG00000079112-CDH17). B) PCA of the combined liver metastasis dataset after adjustment for liver background expression. Density plots indicate the sample distribution along each of the two first components.


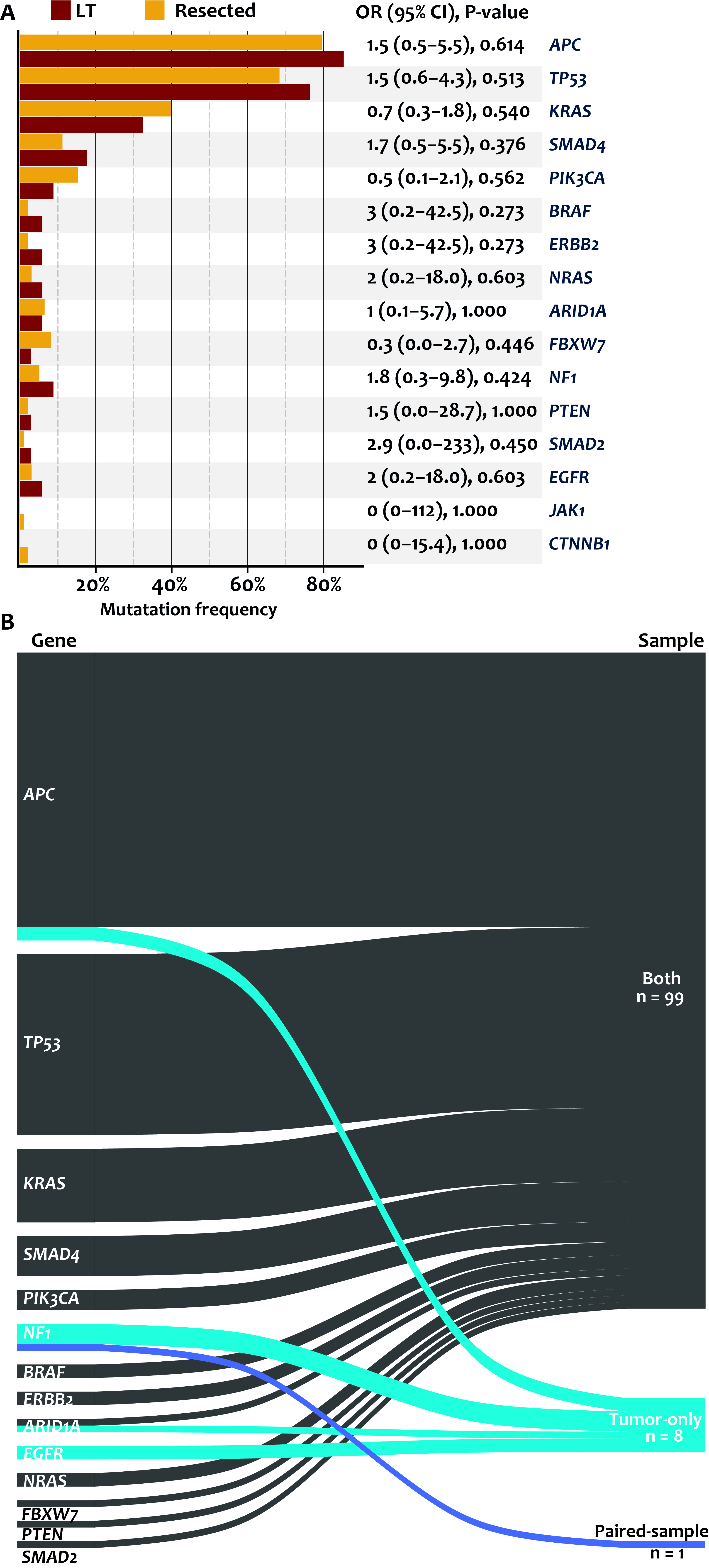


**Figure S4.** **Mutation distribution in patients treated by LT and liver resection.** A) Bar plot of mutation frequencies in the LT (n = 34) and resection cohorts (n = 98). Odds ratios (OR), 95% confidence intervals (CIs) and P-values from Fisher’s exact test of mutation frequencies between the cohorts are indicated. Mutations were classified as somatic using a computational filtering approach in both cohorts. B) Alluvial plot of somatic mutations detected with the paired tumor-normal approach and computational filtering approach in the LT cohort. Somatic mutations were detected in 14 genes (left), most of which were common to both filtering approaches (both; n = 99). A small subset was detected only in paired analyses with the matched normal sample (paired-sample approach; n = 1) or only by the computational filtering approach (tumor-only; n = 8).


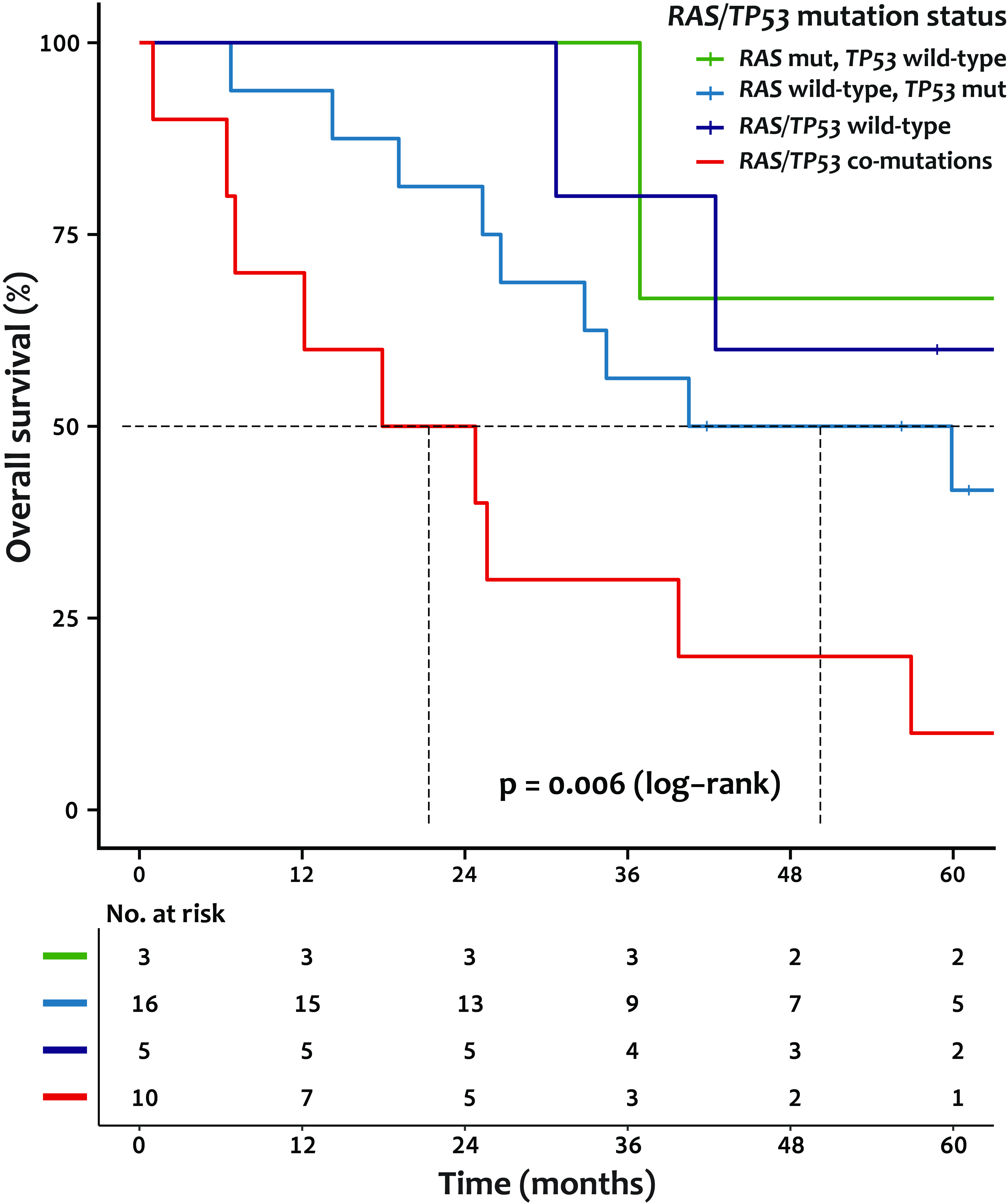


**Figure S5. Overall survival according to RAS and TP53 mutation status.** Kaplan-Meier plot of overall survival according to *RAS* and *TP53* mutations in patients treated by LT (n = 34).





**Figure S6. Overall survival of patients treated by liver transplantation (n = 34) according to RAS/TP53 co-mutations and stratified by clinicopathological variables.** High-risk and low-risk subgroups for each clinicopathological factor are plotted to the left and right, respectively. The red dashed line indicates the survival across all patients in each clinicopathological subgroup and was included for visualization purposes only (not included in statistical analyses). P-values are from log-rank test.


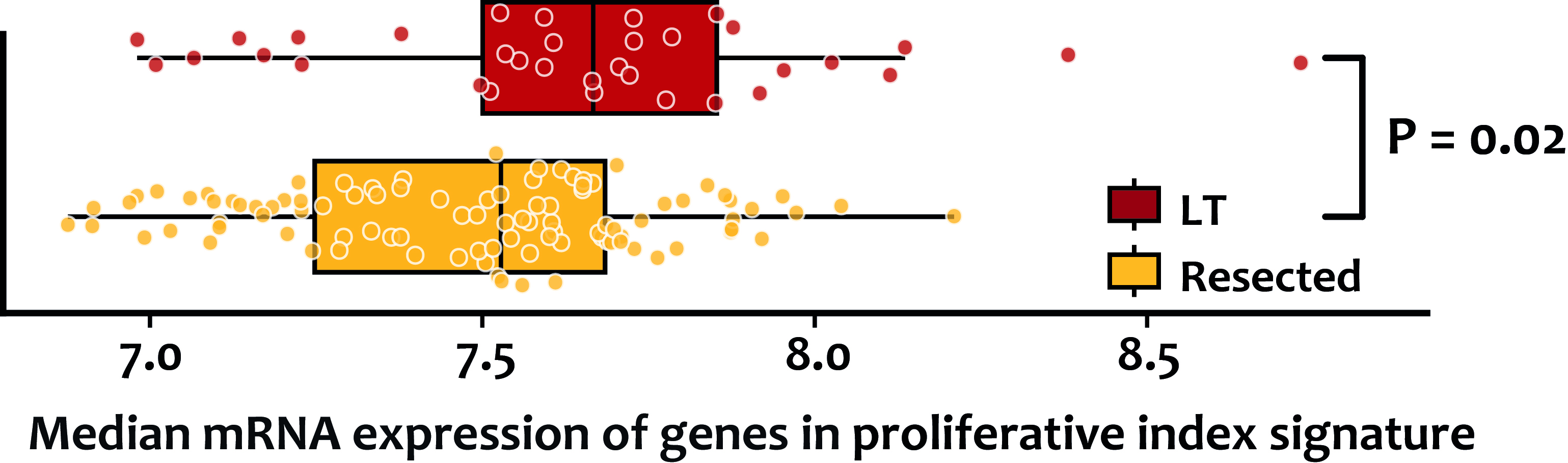


**Figure S7.** **Transcriptomic proliferative index of the LT (n = 34) and resection cohorts (n = 98).** Horizontal boxplot of the proliferative index^15^ according to cohort. Each dot represents one tumor. P-value is from Wilcoxon rank-sum test.


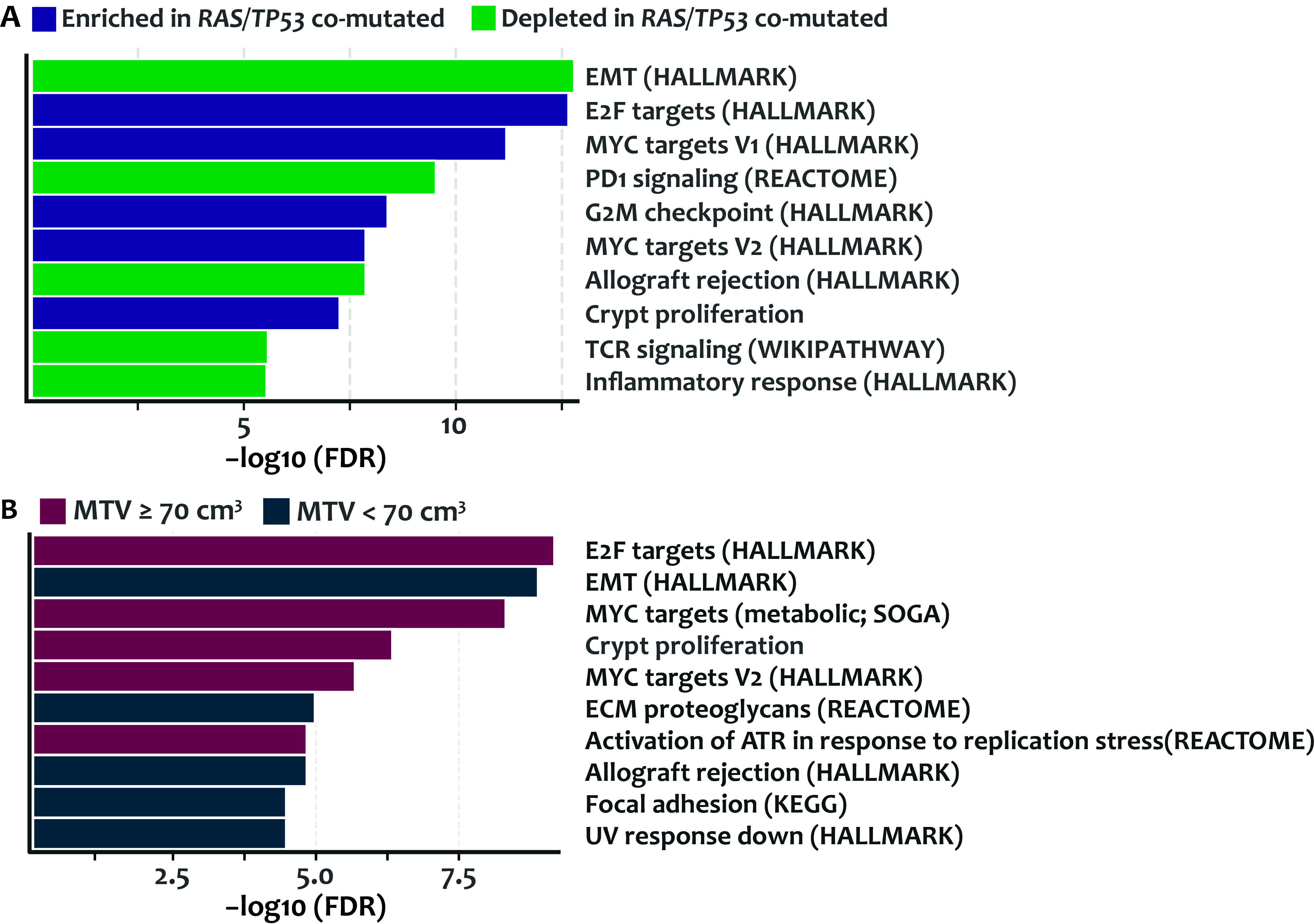


**Figure S8. Gene set enrichment analysis of the LT cohort (n = 34).** Ten most significantly enriched signatures from gene set expression enrichment analyses of a custom gene set collection (n = 260) according to A) *RAS*/*TP53* co-mutation status and B) metabolic tumor volume (MTV) dichotomized at 70 cm^3^ (above: n = 16); below n = 18). P-values are FDR-corrected and plotted on log10-scale. EMT, epithelial-mesenchymal transition; ECM, extracellular matrix; TCR signaling, T cell receptor signaling.

### Table S1. Overview of the custom gene panel for DNA sequencing

| **Gene** | **Coverage** | **Mutation types analyzed** | **Sequencing depth (median, 95% CI)** | |
| --- | --- | --- | --- | --- |
|  |  |  | **LT (n = 34)** | **Liver resection (n = 98)** |
| *APC* | All coding regions | SNVs, indels | (604, 576 - 629) | (529, 514 - 569) |
| *ARID1A* | All coding regions | SNVs, indels | (586, 563 - 607) | (498, 493 - 552) |
| *BRAF* | All coding regions | SNVs, indels | (580, 554 - 611) | (546, 518 - 568) |
| *CCND2* | All coding regions, selected intronic | SNVs, indels, amplifications | (580, 553 - 629) | (551, 532 - 589) |
| *CTNNB1* | All coding regions | SNVs, indels | (586, 573 - 617) | (524, 508 - 561) |
| *EGFR* | All coding regions, selected intronic | SNVs, indels, amplifications | (689, 666 - 738) | (655, 637 - 704) |
| *ERBB2* | All coding regions, selected intronic | SNVs, indels, amplifications | (662, 635 - 698) | (675, 636 - 718) |
| *FBXW7* | All coding regions | SNVs, indels | (488, 474 - 528) | (441, 428 - 473) |
| *JAK1* | All coding regions | SNVs, indels | (546, 529 - 574) | (488, 475 - 529) |
| *KRAS* | All coding regions | SNVs, indels | (450, 437 - 498) | (441, 412 - 454) |
| *MDM2* | All coding regions, selected intronic | SNVs, indels, amplifications | (540, 521 - 592) | (540, 503 - 547) |
| *MYC* | All coding regions, selected intronic | SNVs, indels, amplifications | (818, 794 - 946) | (810, 766 - 870) |
| *NF1* | All coding regions | SNVs, indels | (534, 499 - 557) | (498, 477 - 529) |
| *NRAS* | All coding regions | SNVs, indels | (534, 506 - 553) | (488, 464 - 516) |
| *PIK3CA* | All coding regions | SNVs, indels | (508, 493 - 556) | (478, 460 - 508) |
| *POLE* | Mutation hotspots  (S459F; A456P; M444K; P436R; L424I; V411L; D368Y; F367S; S297F; M295R) | SNVs, indels | (592, 569 - 646) | (586, 575 - 635) |
| *PTEN* | All coding regions | SNVs, indels | (514, 464 - 524) | (469, 442 - 484) |
| *SMAD2* | All coding regions | SNVs, indels | (450, 407 - 469) | (391, 374 - 421) |
| *SMAD4* | All coding regions | SNVs, indels | (450, 392 - 459) | (391, 371 - 421) |
| *TP53* | All coding regions | SNVs, indels | (540, 511 - 582) | (498, 480 - 541) |

### Table S2. Baseline characteristics of the patient cohorts

| **Variables** | **Liver transplantation** | | | **Liver resection** | |
| --- | --- | --- | --- | --- | --- |
|  | **Included**  **(n = 34)**  **no. (%)** | **Excluded (n = 14)**  **no. (%)** | **P-value** | **Patients (n = 98)**  **no. (%)** | **P-value**  **(LT vs. resection)** |
| **Sex** |  |  | 1^a^ |  | 0.8^a^ |
| Male | 20 (59) | 8 (57) |  | 60 (61) |  |
| Female | 14 (41) | 6 (43) |  | 38 (38) |  |
| **Age** |  |  | 0.8^a^ |  | 0.002^a^ |
| Above median (≥57) | 17 (50) | 8 (57) |  | 78 (80) |  |
| Below median (< 57) | 17 (50) | 6 (43) |  | 20 (20) |  |
| **Primary tumor T stage** |  |  | 0.4^b^ |  | 0.1^b^ |
| T0 | 2 (6) | 1 (7.1) |  | 0 |  |
| T1 | 0 (0) | 1 (7.1) |  | 1 (1) |  |
| T2 | 3 (9) | 2 (14.3) |  | 6 (6) |  |
| T3 | 25 (73) | 10 (71.4) |  | 71 (72) |  |
| T4 | 4 (12) | 0 |  | 18 (18) |  |
| Unknown | 0 | 0 |  | 2 (2) |  |
| **Primary tumor N stage** |  |  | 0.1^b^ |  | 0.4^b^ |
| N0 | 11 (32) | 9 (64) |  | 28 (28.6) |  |
| N1 | 9 (26) | 3 (21) |  | 38 (38.8) |  |
| N2 | 14 (41) | 2 (14) |  | 31 (31.6) |  |
| Unknown | 0 | 0 |  | 1 (1) |  |
| **Primary tumor location** |  |  | 0.3^b^ |  | 0.7^b^ |
| Right | 9 (27) | 1 (7) |  | 23 (23) |  |
| Left | 13 (38) | 7 (50) |  | 47 (48) |  |
| Rectum | 11 (32) | 6 (43) |  | 27 (28) |  |
| Unknown | 1 (3) | 0 |  | 1 (1) |  |
| **Number of liver metastases** |  |  | 0.7^a^ |  | 0.004^a^ |
| ≥ 9 | 17 (50) | 5 (36) |  | 21 (21) |  |
| < 9 | 17 (50) | 7 (50) |  | 77 (79) |  |
| Unknown | 0 | 2 (14) |  | 0 |  |
| **Size of largest liver metastasis (mm)** |  |  | 0.5^a^ |  | 0.5^a^ |
| ≥ 55 | 11 (32) | 2 (14) |  | 25 (26) |  |
| < 55 | 23 (68) | 10 (72) |  | 72 (74) |  |
| Unknown | 0 | 2 (14) |  | 1 (1) |  |
| **Lines of chemotherapy prior to surgery** |  |  | 0.5^b^ |  |  |
| 1 | 11 (33) | 7 (50) |  | na^c^ |  |
| 2 | 17 (52) | 6 (43) |  | na^c^ |  |
| 3 | 5 (15) | 1 (7) |  | na^c^ |  |
| Unknown | 1 | 0 |  |  |  |
| ***RAS* (*KRAS/NRAS*)** |  |  | - |  | 0.7^a^ |
| Mutated | 13 (38) | - |  | 42 (43) |  |
| Wild-type | 21 (62) | - |  | 56 (57) |  |
| ***RAS/TP53*** |  |  | - |  | 0.7^a^ |
| Mutated | 10 (29) | - |  | 25 (26) |  |
| Wild-type | 24 (71) | - |  | 73 (74) |  |
| **Carcinoembryonic antigen (µg/mL)** |  |  | 0.2^a^ |  |  |
| ≥ 80 | 9 (26.5) | 1 (7) |  | na |  |
| < 80 | 25 (73.5) | 13 (93) |  | na |  |
| **Metabolic tumor volume (cm³)** |  |  | 0.05^a^ |  |  |
| ≥ 70 | 16 (47) | 2 (14) |  | na |  |
| < 70 | 18 (53) | 12 (86) |  | na |  |
| ^a^Fisher's exact test, excluding unknown values; ^b^Pearson's χ^2^-test, excluding unknown values; ^c^All patients had at least 1 line of chemotherapy | | | | | |

### Table S3. Detected mutations in the LT and resection cohorts

The table is included in a separate file and is accessible from https://doi.org/10.5281/zenodo.14982972.

### Table S4. Comparison of mutation frequencies between the external dataset of biopsies from unresected liver metastases (MSK; n = 73)^27^ and LT cohort (n = 34)

| **Gene** | **Fisher's test** | | | | **Counts** | | | |
| --- | --- | --- | --- | --- | --- | --- | --- | --- |
|  | **Odds ratio** | **95% CI** | **P-value** | **FDR-corrected P-value (Benjamini-Hochberg)** | **Mutated (MSK)** | **Mutated (LT)** | **Wild-type (MSK)** | **Wild-type (LT)** |
| *APC* | 0.8 | 0.22 - 2.35 | 0.798 | 1.0 | 57 | 28 | 16 | 6 |
| *TP53* | 1.6 | 0.49 - 4.73 | 0.429 | 1.0 | 61 | 26 | 12 | 8 |
| *KRAS* | 1.5 | 0.61 - 4.04 | 0.397 | 1.0 | 31 | 11 | 42 | 23 |
| *NRAS* | 0.9 | 0.13 - 10.76 | 1.000 | 1.0 | 4 | 2 | 69 | 32 |
| *RAS* | 1.5 | 0.6 - 3.75 | 0.407 | 1.0 | 35 | 13 | 38 | 21 |
| *SMAD4* | 0.8 | 0.25 - 3.02 | 0.780 | 1.0 | 11 | 6 | 62 | 28 |
| *PIK3CA* | 2.4 | 0.61 - 14.2 | 0.257 | 1.0 | 14 | 3 | 59 | 31 |
| *BRAF* | 3.1 | 0.63 - 30.4 | 0.217 | 1.0 | 12 | 2 | 61 | 32 |
| *ERBB2* | 0.7 | 0.07 - 8.62 | 0.652 | 1.0 | 3 | 2 | 70 | 32 |
| *ARID1A* | 1.4 | 0.11 - 76.44 | 1.000 | 1.0 | 3 | 1 | 70 | 33 |
| *FBXW7* | 1.9 | 0.18 - 96.98 | 1.000 | 1.0 | 4 | 1 | 69 | 33 |
| *NF1* | 0.9 | 0.05 - 56.47 | 1.000 | 1.0 | 2 | 1 | 71 | 33 |
| *PTEN* | 0.9 | 0.05 - 56.47 | 1.000 | 1.0 | 2 | 1 | 71 | 33 |
| *SMAD2* | 0.5 | 0.01 - 37.06 | 0.537 | 1.0 | 1 | 1 | 72 | 33 |
| *CTNNB1* | Inf | 0.43 - Inf | 0.176 | 1.0 | 5 | 0 | 68 | 34 |
| *EGFR* | NA | NA | NA | NA | 0 | 0 | 73 | 34 |
| *JAK1* | NA | NA | NA | NA | 0 | 0 | 73 | 34 |

### Table S5. Selected clinicopathological variables according to mutation status of the most frequently mutated genes among patients in the LT cohort (n = 34)

| **Variable** | **Mutation** | **Odds ratio** | **95% CI** | **P-value (Fisher's test)** | **FDR-corrected P-value (Benjamini-Hochberg)** |
| --- | --- | --- | --- | --- | --- |
| **Sex (male versus female)** | | | | | |
|  | *APC* | 0.7 | 0.1 - 5.7 | 1.000 | 1.000 |
|  | *TP53* | 0.4 | 0 - 2.8 | 0.422 | 0.993 |
|  | *KRAS* | 0.8 | 0.1 - 4.3 | 1.000 | 1.000 |
|  | *NRAS* | 0.7 | 0 - 58 | 1.000 | 1.000 |
|  | *RAS* | 0.7 | 0.1 - 3.7 | 0.728 | 1.000 |
|  | *SMAD4* | 1.5 | 0.2 - 19 | 1.000 | 1.000 |
|  | *RAS/TP53* | 0.6 | 0.1 - 3.5 | 0.704 | 1.000 |
| **Size of largest liver metastasis (above versus below 55 mm)^a^** | | | | | |
|  | *APC* | Inf | 0.6 - Inf | 0.145 | 0.611 |
|  | *TP53* | Inf | 1 - Inf | 0.034 | 0.309 |
|  | *KRAS* | 1.3 | 0.2 - 7.4 | 1.000 | 1.000 |
|  | *NRAS* | 2.1 | 0 - 180 | 1.000 | 1.000 |
|  | *RAS* | 1.5 | 0.3 - 8.5 | 0.709 | 1.000 |
|  | *SMAD4* | 1.1 | 0.1 - 9.1 | 1.000 | 1.000 |
|  | *RAS/TP53* | 2.9 | 0.5 - 18.2 | 0.232 | 0.749 |
| **Number of liver metastases (above versus below 9)^a^** | | | | | |
|  | *APC* | 1.0 | 0.1 - 8.8 | 1.000 | 1.000 |
|  | *TP53* | 1.0 | 0.1 - 6.7 | 1.000 | 1.000 |
|  | *KRAS* | 1.3 | 0.2 - 7.1 | 1.000 | 1.000 |
|  | *NRAS* | 1.0 | 0 - 83.3 | 1.000 | 1.000 |
|  | *RAS* | 1.3 | 0.3 - 6.4 | 1.000 | 1.000 |
|  | *SMAD4* | 6.3 | 0.6 - 333.9 | 0.175 | 0.612 |
|  | *RAS/TP53* | 1.7 | 0.3 - 11 | 0.708 | 1.000 |
| **Carcinoembryonic antigen level (above versus below 80 µg/L)^a^** | | | | | |
|  | *APC* | Inf | 0.4 - Inf | 0.162 | 0.612 |
|  | *TP53* | Inf | 0.7 - Inf | 0.077 | 0.404 |
|  | *KRAS* | 2.0 | 0.3 - 13 | 0.425 | 0.993 |
|  | *NRAS* | 0.0 | 0 - 15.2 | 1.000 | 1.000 |
|  | *RAS* | 1.4 | 0.2 - 8.6 | 0.704 | 1.000 |
|  | *SMAD4* | 0.5 | 0 - 5.7 | 1.000 | 1.000 |
|  | *RAS/TP53* | 2.5 | 0.4 - 16.3 | 0.395 | 0.993 |
| **Metabolic tumor volume (above versus below 70 cm3)^a^** | | | | | |
|  | *APC* | Inf | 1.2 - Inf | 0.020 | 0.277 |
|  | *TP53* | 9.0 | 0.9 - 457 | 0.043 | 0.309 |
|  | *KRAS* | 1.5 | 0.3 - 8.5 | 0.717 | 1.000 |
|  | *NRAS* | 1.1 | 0 - 94 | 1.000 | 1.000 |
|  | *RAS* | 1.5 | 0.3 - 7.8 | 0.725 | 1.000 |
|  | *SMAD4* | 2.6 | 0.3 - 33.2 | 0.387 | 0.993 |
|  | *RAS/TP53* | 3.7 | 0.6 - 28.2 | 0.134 | 0.611 |
| **Primary tumor location (right versus left or rectum)^a^** | | | | | |
|  | *APC* | 0.7 | 0.1 - 9.5 | 1.000 | 1.000 |
|  | *TP53* | Inf | 0.7 - Inf | 0.073 | 0.404 |
|  | *KRAS* | 9.1 | 1.3 - 83.5 | 0.010 | 0.220 |
|  | *NRAS* | 0.0 | 0 - 14.6 | 1.000 | 1.000 |
|  | *RAS* | 5.6 | 0.9 - 46.4 | 0.044 | 0.309 |
|  | *SMAD4* | 3.3 | 0.3 - 32.3 | 0.309 | 0.928 |
|  | *RAS/TP53* | 12.4 | 1.7 - 129.1 | 0.005 | 0.196 |
| ^a^Variables were categorized according to thresholds defined in Dueland et al., JAMA Surg 2023;158(9)e232932 | | | | | |

### Table S6. Univariable Cox proportional hazards analyses of OS among patients in the LT cohort (n = 34)

| **Variable** | **Categories^a^** | **HR** | **P-value (Wald test)** | **FDR-corrected P-value (Benjamini-Hochberg)** | **95% CI** |
| --- | --- | --- | --- | --- | --- |
| *KRAS* | Wild-type (ref) vs. mutated | 2.3 | 0.053 | 0.140 | 1-5.1 |
| *RAS* | Wild-type (ref) vs. mutated | 2.2 | 0.057 | 0.140 | 1-4.9 |
| *TP53* | Wild-type (ref) vs. mutated | 2.9 | 0.062 | 0.140 | 1-8.6 |
| *RAS/TP53* | Single or no mutation (ref) vs. co-mutation | 4.0 | 0.002 | 0.018 | 1.7-9.3 |
| *APC* | Wild-type (ref) vs. mutated | 1.7 | 0.365 | 0.483 | 0.5-5.9 |
| *APC/PIK3CA* | Single or no mutation (ref) vs. co-mutation | 2.4 | 0.242 | 0.335 | 0.6-11 |
| *SMAD4* | Wild-type (ref) vs. mutated | 1.9 | 0.198 | 0.314 | 0.7-5.2 |
| Sex | Male (ref) vs. female | 2.2 | 0.074 | 0.148 | 0.9-5.4 |
| Age | Above (ref) vs. below median (57 years) | 1.2 | 0.639 | 0.677 | 0.5-2.7 |
| MTV | Below (ref) vs. above 70 cm3^a^ | 2.5 | 0.028 | 0.101 | 1.1-5.7 |
| CEA | Below (ref) vs. above 80 µg/mL^a^ | 3.7 | 0.004 | 0.024 | 1.5-8.9 |
| Size of largest liver metastasis (mm) | Below (ref) vs. above 55 cm^a^ | 3.1 | 0.010 | 0.045 | 1.3-7.4 |
| No. of liver metastases | Below (ref) vs. above 9^a^ | 2.0 | 0.092 | 0.166 | 0.9-4.6 |
| Primary tumor site | Left or rectum (ref) vs. right | 4.3 | 0.001 | 0.018 | 1.8-10 |
| Primary T stage | T0 (ref) vs. T2 | 0.5 | 0.501 | 0.564 | 0.08-3.4 |
|  | T0 (ref) vs. T3 | 0.3 | 0.107 | 0.175 | 0.06-1.3 |
|  | T0 (ref) vs. T4 | 0.4 | 0.381 | 0.490 | 0.07-2.7 |
| Primary N stage | N0 (ref) vs. N1 | 1.1 | 0.824 | 0.824 | 0.4-3.4 |
|  | N0 (ref) vs. N2 | 1.5 | 0.416 | 0.499 | 0.6-3.8 |
| ^a^Variables were categorized according to thresholds defined in Dueland et al., JAMA Surg 2023;158(9)e232932.  All variables complied with the proportional hazards assumption according to the scaled Schoenfeld residuals. | | | | | |

### Table S7. Bivariable Cox proportional hazards analyses for *RAS*/*TP53* co-mutations and selected variables among patients in the LT cohort (n = 34)

| **Variable** | **Categories^a^** | **HR** | **95% CI** | **P-value (Wald test)** |
| --- | --- | --- | --- | --- |
| ***RAS/TP53* + MTV** |  |  |  |  |
| *RAS/TP53* | Single or no mutation (ref) vs. co-mutation | 3.3 | 1.3 - 7.9 | 0.009 |
| MTV | Above vs. below 70 cm3 (ref) | 1.9 | 0.82 - 4.5 | 0.134 |
| Global test |  |  |  | 0.002 |
| ***RAS/TP53* + CEA** |  |  |  |  |
| *RAS/TP53* | Single or no mutation (ref) vs. co-mutation | 3.5 | 1.5 - 8.4 | 0.004 |
| CEA high | Above vs. below 80 µg/mL (ref) | 3.2 | 1.3 - 8 | 0.0103 |
| Global test |  |  |  | 0.0003 |
| ***RAS/TP53* + size of liver metastases** | |  |  |  |
| *RAS/TP53* | Single or no mutation (ref) vs. co-mutation | 3.1 | 1.2 - 7.6 | 0.014 |
| Size | Above vs. below 55 cm (ref) | 2.2 | 0.89 - 5.4 | 0.089 |
| Global test |  |  |  | 0.001 |
| ***RAS/TP53* + no. of liver metastases** | |  |  |  |
| *RAS/TP53* | Single or no mutation (ref) vs. co-mutation | 3.6 | 1.5 - 8.5 | 0.004 |
| Number | Above vs. below 9 (ref) | 1.7 | 0.73 - 3.9 | 0.222 |
| Global test |  |  |  | 0.003 |
| ***RAS/TP53* + primary tumor location** | |  |  |  |
| *RAS/TP53* | Single or no mutation (ref) vs. co-mutation | 2.7 | 1 - 6.8 | 0.043 |
| Location | Right vs. left or rectum (ref) | 3.2 | 1.3 - 8.3 | 0.013 |
| Global test |  |  |  | 0.0007 |
| ^a^Variables were categorized according to thresholds defined in Dueland et al., JAMA Surg 2023;158(9)e232932  All variables complied with the proportional hazards assumption according to the scaled Schoenfeld residuals. | | | | |

### Table S8. Differentially expressed genes (FDR-corrected P-value < 0.05) between patients in the resection (n = 98) versus LT cohort (n = 34)

The table is included in a separate file and is accessible from https://doi.org/10.5281/zenodo.14982972.

### Table S9. Top 10 over-represented pathways in the Reactome database (ranked by statistical significance) for differentially expressed genes between the resection (n = 98) and LT cohorts (n = 34)^a^

| **Reactome pathway ID** | **Description** | **Cohort** | **FDR (P-value hypergeometric test)** | **q-value** |
| --- | --- | --- | --- | --- |
| R-HSA-176187 | Activation of ATR in response to replication stress | LT | 3E-14 | 2E-14 |
| R-HSA-69052 | Switching of origins to a post-replicative state | LT | 1E-13 | 9E-14 |
| R-HSA-9675135 | Diseases of DNA repair | LT | 7E-13 | 5E-13 |
| R-HSA-5693579 | Homologous DNA Pairing and Strand Exchange | LT | 2E-12 | 1E-12 |
| R-HSA-9675136 | Diseases of DNA Double-Strand Break Repair | LT | 3E-12 | 2E-12 |
| R-HSA-9701190 | Defective homologous recombination repair (HRR) due to BRCA2 loss of function | LT | 3E-12 | 2E-12 |
| R-HSA-5693616 | Presynaptic phase of homologous DNA pairing and strand exchange | LT | 1E-11 | 1E-11 |
| R-HSA-5685942 | HDR through Homologous Recombination (HRR) | LT | 3E-11 | 2E-11 |
| R-HSA-68949 | Orc1 removal from chromatin | LT | 5E-11 | 3E-11 |
| R-HSA-68962 | Activation of the pre-replicative complex | LT | 6E-11 | 4E-11 |
| R-HSA-877300 | Interferon gamma signaling | resection | 7E-05 | 6E-05 |
| R-HSA-216083 | Integrin cell surface interactions | resection | 1E-04 | 1E-04 |
| R-HSA-202433 | Generation of second messenger molecules | resection | 2E-04 | 2E-04 |
| R-HSA-2022090 | Assembly of collagen fibrils and other multimeric structures | resection | 1E-03 | 1E-03 |
| R-HSA-512988 | Interleukin-3, Interleukin-5 and GM-CSF signaling | resection | 1E-03 | 1E-03 |
| R-HSA-451927 | Interleukin-2 family signaling | resection | 1E-03 | 1E-03 |
| R-HSA-1442490 | Collagen degradation | resection | 1E-03 | 1E-03 |
| R-HSA-6783783 | Interleukin-10 signaling | resection | 1E-03 | 1E-03 |
| R-HSA-1474290 | Collagen formation | resection | 3E-03 | 3E-03 |
| R-HSA-1980143 | Signaling by NOTCH1 | resection | 3E-03 | 3E-03 |
| ^a^Analysis using n = 2 000 upregulated genes in each cohort ranked by log2 fold-change and with FDR-corrected P < 0.05. | | | | |

### Table S10. Gene set enrichment analyses (GSEA) of sample groups

The table is included in a separate file and is accessible from https://doi.org/10.5281/zenodo.14982972.

# References

1. Dueland S, Smedman TM, Syversveen T, Grut H, Hagness M, Line P-D. Long-Term Survival, Prognostic Factors, and Selection of Patients With Colorectal Cancer for Liver Transplant: A Nonrandomized Controlled Trial. *JAMA Surg*. 2023;158:e232932.

2. Dueland S, Grut H, Syversveen T, Hagness M, Line P-D. Selection criteria related to long-term survival following liver transplantation for colorectal liver metastasis. *Am J Transplant*. 2020;20:530–537.

3. Dueland S, Smedman TM, Røsok B, Grut H, Syversveen T, Jørgensen LH, et al. Treatment of relapse and survival outcomes after liver transplantation in patients with colorectal liver metastases. *Transpl Int*. 2021;34:2205–2213.

4. Grut H, Solberg S, Seierstad T, Revheim ME, Egge TS, Larsen SG, et al. Growth rates of pulmonary metastases after liver transplantation for unresectable colorectal liver metastases. *Br J Surg*. 2018;105:295–301.

5. Brunsell TH, Cengija V, Sveen A, Bjørnbeth BA, Røsok BI, Brudvik KW, et al. Heterogeneous radiological response to neoadjuvant therapy is associated with poor prognosis after resection of colorectal liver metastases. *Eur J Surg Oncol*. 2019;45:2340–2346.

6. Brunsell TH, Sveen A, Bjørnbeth BA, Røsok BI, Danielsen SA, Brudvik KW, et al. High Concordance and Negative Prognostic Impact of RAS/BRAF/PIK3CA Mutations in Multiple Resected Colorectal Liver Metastases. *Clin Colorectal Cancer*. 2020;19:e26–e47.

7. Moosavi SH, Eide PW, Eilertsen IA, Brunsell TH, Berg KCG, Røsok BI, et al. De novo transcriptomic subtyping of colorectal cancer liver metastases in the context of tumor heterogeneity. *Genome Med*. 2021;13:143.

8. Eide PW, Moosavi SH, Eilertsen IA, Brunsell TH, Langerud J, Berg KCG, et al. Metastatic heterogeneity of the consensus molecular subtypes of colorectal cancer. *Npj Genomic Med*. 2021;6:59.

9. Dai M. Evolving gene/transcript definitions significantly alter the interpretation of GeneChip data. *Nucleic Acids Res*. 2005;33:e175–e175.

10. Gautier L, Cope L, Bolstad BM, Irizarry RA. affy—analysis of *Affymetrix GeneChip* data at the probe level. *Bioinformatics*. 2004;20:307–315.

11. Durinck S, Spellman PT, Birney E, Huber W. Mapping identifiers for the integration of genomic datasets with the R/Bioconductor package biomaRt. *Nat Protoc*. 2009;4:1184–1191.

12. Ritchie ME, Phipson B, Wu D, Hu Y, Law CW, Shi W, et al. limma powers differential expression analyses for RNA-sequencing and microarray studies. *Nucleic Acids Res*. 2015;43:e47–e47.

13. Milacic M, Beavers D, Conley P, Gong C, Gillespie M, Griss J, et al. The Reactome Pathway Knowledgebase 2024. *Nucleic Acids Res*. 2024;52:D672–D678.

14. Hänzelmann S, Castelo R, Guinney J. GSVA: gene set variation analysis for microarray and RNA-Seq data. *BMC Bioinformatics*. 2013;14:7.

15. Ramaker RC, Lasseigne BN, Hardigan AA, Palacio L, Gunther DS, Myers RM, et al. RNA sequencing-based cell proliferation analysis across 19 cancers identifies a subset of proliferation-informative cancers with a common survival signature. *Oncotarget*. 2017;8:38668–38681.

16. Sveen A, Bruun J, Eide PW, Eilertsen IA, Ramirez L, Murumägi A, et al. Colorectal Cancer Consensus Molecular Subtypes Translated to Preclinical Models Uncover Potentially Targetable Cancer Cell Dependencies. *Clin Cancer Res*. 2018;24:794–806.

17. Chaisaingmongkol J, Budhu A, Dang H, Rabibhadana S, Pupacdi B, Kwon SM, et al. Common Molecular Subtypes Among Asian Hepatocellular Carcinoma and Cholangiocarcinoma. *Cancer Cell*. 2017;32:57-70.e3.

18. Uhlén M, Fagerberg L, Hallström BM, Lindskog C, Oksvold P, Mardinoglu A, et al. Tissue-based map of the human proteome. *Science*. 2015;347:1260419.

19. Li H, Durbin R. Fast and accurate short read alignment with Burrows–Wheeler transform. *Bioinformatics*. 2009;25:1754–1760.

20. McKenna A, Hanna M, Banks E, Sivachenko A, Cibulskis K, Kernytsky A, et al. The Genome Analysis Toolkit: A MapReduce framework for analyzing next-generation DNA sequencing data. *Genome Res*. 2010;20:1297–1303.

21. Cibulskis K, Lawrence MS, Carter SL, Sivachenko A, Jaffe D, Sougnez C, et al. Sensitive detection of somatic point mutations in impure and heterogeneous cancer samples. *Nat Biotechnol*. 2013;31:213–219.

22. Wang K, Li M, Hakonarson H. ANNOVAR: functional annotation of genetic variants from high-throughput sequencing data. *Nucleic Acids Res*. 2010;38:e164–e164.

23. The 1000 Genomes Project Consortium, Corresponding authors, Auton A, Abecasis GR, Steering committee, Altshuler DM, et al. A global reference for human genetic variation. *Nature*. 2015;526:68–74.

24. Sherry ST. dbSNP: the NCBI database of genetic variation. *Nucleic Acids Res*. 2001;29:308–311.

25. Tate JG, Bamford S, Jubb HC, Sondka Z, Beare DM, Bindal N, et al. COSMIC: the Catalogue Of Somatic Mutations In Cancer. *Nucleic Acids Res*. 2019;47:D941–D947.

26. Cerami E, Gao J, Dogrusoz U, Gross BE, Sumer SO, Aksoy BA, et al. The cBio Cancer Genomics Portal: An Open Platform for Exploring Multidimensional Cancer Genomics Data. *Cancer Discov*. 2012;2:401–404.

27. Yaeger R, Chatila WK, Lipsyc MD, Hechtman JF, Cercek A, Sanchez-Vega F, et al. Clinical Sequencing Defines the Genomic Landscape of Metastatic Colorectal Cancer. *Cancer Cell*. 2018;33:125-136.e3.

28. Smedman TM, Line P-D, Hagness M, Syversveen T, Grut H, Dueland S. Liver transplantation for unresectable colorectal liver metastases in patients and donors with extended criteria (SECA-II arm D study). *BJS Open*. 2020;4:467–477.
